# The Paxillin MoPax1 Activates Mitogen-Activated Protein (MAP) Kinase Signaling Pathways and Autophagy through MAP Kinase Activator MoMka1 during Appressorium-Mediated Plant Infection by the Rice Blast Fungus Magnaporthe oryzae

**DOI:** 10.1128/mbio.02218-22

**Published:** 2022-10-31

**Authors:** Wuyun Lv, Yu Xiao, Zhe Xu, Hong Jiang, Qi Tong, Zhengyi Wang

**Affiliations:** a State Key Laboratory of Rice Biology and Ministry of Agriculture Key Laboratory of Molecular Biology of Crop Pathogens and Insects, Institute of Biotechnology, Zhejiang University, Hangzhou, China; b College of Tea Science and Tea Culture/Collaborative Innovation Center for Efficient and Green Production of Agriculture in Mountainous Areas of Zhejiang Province, Zhejiang A&F University, Hangzhou, China; Universidade de São Paulo

**Keywords:** *Magnaporthe oryzae*, MoMka1, MoPax1, appressoria, autophagy, mitogen-activated protein kinases

## Abstract

Paxillin is a focal adhesion-associated protein that functions as an adaptor to recruit diverse cytoskeleton and signaling molecules into a complex and plays a crucial role in several signaling pathways in mammal cells. However, paxillin-mediated signal pathways are largely unknown in phytopathogenic fungi. Previously, Pax1 of Magnaporthe oryzae (MoPax1), a paxillin-like protein, has been identified as a crucial pathogenicity determinant. Here, we report the identification of a mitogen-activated protein (MAP) kinase (MAPK) activator, Mka1 of M. oryzae (MoMka1), that physically interacts with MoPax1. Targeted gene deletion of *MoMKA1* resulted in pleiotropic defects in aerial hyphal growth, conidiation, appressorium formation, and pathogenicity in M. oryzae. MoMka1 interacts with Mst50, an adaptor protein of the Mst11-Mst7-Pmk1 and Mck1-Mkk2-Mps1 cascades. Moreover, the phosphorylation levels of both Pmk1 and Mps1 in aerial hyphae of the *ΔMomka1* mutant were significantly reduced, indicating that MoMka1 acts upstream from the MAPK pathways. Interestingly, we found that MoMka1 interacts with MoAtg6 and MoAtg13. Deletion of *MoMKA1* led to impaired MoAtg13 phosphorylation and enhanced autophagic flux under nutrient-rich conditions, indicating that MoMka1 is required for regulation of autophagy in M. oryzae. Taken together, the paxillin MoPax1 may activate MAP kinase signaling pathways and autophagy through MAP kinase activator MoMka1 and play important roles during appressorium-mediated plant infection by the rice blast fungus.

## INTRODUCTION

The ascomycetous fungus Magnaporthe oryzae is the agent that causes rice blast disease in cultivated rice globally and, more recently, in wheat in South America and Bangladesh (1, 2). The rice blast disease cycle starts when a three-celled conidium is released from conidiophores and attaches to rice leaves. The conidium rapidly germinates and, at the tip of the germ tube, forms a dome-shaped specialized infection structure called an appressorium (3). The appressorium becomes highly melanized as it matures and generates enormous turgor, which is applied as physical force to penetrate the plant cuticle (4). By 3 to 5 days after penetration, the fungus colonizes host cells and produces a visible necrotic lesion on the rice leaf surface (5, 6). New conidia are generated from aerial conidiophores that differentiate from hyphae within the lesion. Eventually, these conidia are released and dispersed, providing the inoculum for next infection cycle.

In the past 3 decades, considerable efforts have been made in understanding the molecular mechanisms that regulate appressorium morphogenesis and penetration in M. oryzae. It is clear that appressorium maturation and invasive growth are regulated by the Pmk1 mitogen-activated protein (MAP) kinase (Pmk1-MAPK) pathway (7, 8). The other MAP kinase pathway, Mps1-MAPK, is required for appressorium-mediated penetration but dispensable for appressorium formation (9). Interestingly, Mst50 can act as an adaptor or scaffold protein for both the Mst11-Mst7 and Mck1-Mkk2 signaling modules of the two MAPK pathways in M. oryzae (10, 11). Moreover, numerous Mst50-interacting proteins, including Ras1/2, Mgb1, and Cdc42, have been identified (10, 11). Apart from the MAPK pathways, autophagy is also important for regulating conidial cell death, appressorium maturation, penetration peg formation, and pathogenicity by M. oryzae (12–15). Recently, considerable progress has been made in exploring cross talk networks between the different signaling pathways and cell processes for regulating fungal development and pathogenicity in M. oryzae (16, 17). However, the upstream components and regulatory mechanisms of the two MAPK signal pathways and autophagy are still not fully understood.

In mammal cells, paxillin plays important roles in several signaling pathways in regulating cell growth, differentiation, proliferation, and motility. Paxillin contains a number of motifs, including LD motifs, LIM domains, and SH3 (Src homology 3) domain-binding sites (18). These motifs that mediate protein-protein interaction are crucial docking sites for cytoskeletal proteins, kinases, GTPase-activating proteins, and other adaptor proteins (18). Therefore, paxillin serves as an adaptor protein, recruiting specific signaling molecules into a complex to coordinate downstream signaling pathways. However, in phytopathogenic fungi, how paxillin mediates signaling pathways in regulating morphogenesis and pathogenicity remains elusive.

In our previous study, we identified a three-LIM-domain-containing paxillin-like protein (Pax1 of M. oryzae [MoPax1]) that plays important roles in hyphal growth, conidiogenesis, appressorium formation, penetration, and pathogenicity in M. oryzae (19). To further investigate roles of MoPax1 in signaling transduction and plant infection, we had been trying to identify partner proteins of MoPax1. By the screening assays, an MAP kinase activator named MoMka1 (Mka1 of M. oryzae), was determined. Targeted gene deletion of *MoMKA1* resulted in pleiotropic defects in aerial hyphal growth, conidiation, appressorium formation, and pathogenicity in M. oryzae. Moreover, MoMka1 interacts with Mst50, and the phosphorylation levels of both Pmk1 and Mps1 in aerial hyphae of the *ΔMomka1* mutants were significantly reduced. Interestingly, MoMka1 interacts with both MoAtg6 and MoAtg13 and is required for regulation of the autophagy process by M. oryzae. Taken together, we conclude that the paxillin MoPax1 activates MAP kinase signaling pathways and autophagy through MAP kinase activator MoMka1 during appressorium-mediated plant infection by the rice blast fungus.

## RESULTS

### MoMka1 interacts with MoPax1.

**FIG 1:**
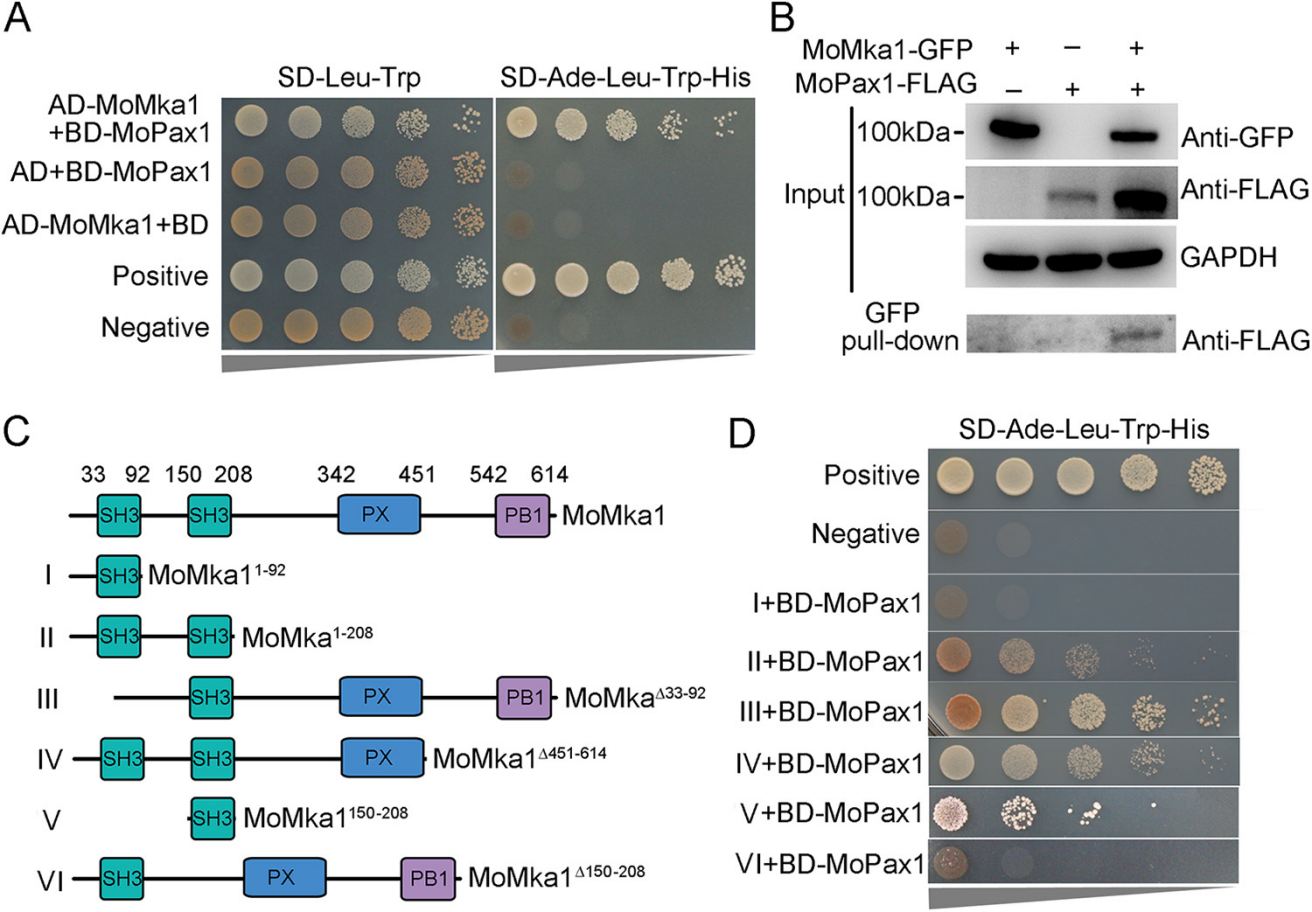
MoMka1 interacts with MoPax1 via the second SH3 domain in M. oryzae. (A) Yeast two-hybrid (Y2H) analysis of the interaction between MoMka1 and MoPax1. The fusion constructs *MoMKA1*-pGADT7 and *MoPAX1*-pGBKT7 were cointroduced into the yeast Y2H Gold strain, and transformants were cultured on synthetic defined medium (SD)-Leu-Trp plates as a control and on SD-Ade-Leu-Trp-His for 3 days. The pair of plasmids pGADT7-T and pGBKT7-53 was used as the positive control, and the pair of plasmids pGADT7-T and pGBKT7-Lam was used as the negative control. AD, the pGADT7 plasmid; BD, the pGBKT7 plasmid. (B) Coimmunoprecipitation (co-IP) assays for the interaction between MoMka1 and MoPax1. The MoMka1-GFP and MoPax1-3×FLAG fusion constructs were coexpressed in the Ku80 strain. The isolated proteins were analyzed by Western blotting using anti-GFP and anti-Flag antibodies. (C) Domain map of MoMka1. The domain prediction of MoMka1 was performed with the SMART analysis service. (D) Y2H assays between four MoMka1 variants and MoPax1. I, only containing the first SH3 domain; II, carrying two SH3 domains; III, lacking the first SH3 domain; IV, lacking the PB1 domain; Ⅴ, only containing the second SH3 domain; Ⅵ, lacking the second SH3 domain. Pairs of different combinations of the truncated constructs of MoMka1 and MoPax1 were cotransformed into Y2H Gold.

To identify MoPax1-interacting proteins, we carried out large-scale screening assays by yeast two-hybrid (Y2H) analysis. Among about 50 candidate proteins, we found that MoPax1 physically interacted with MoMka1 (MGG_01702), an MAP kinase activator (Fig. 1A). To further confirm the interaction between the two proteins, we generated MoMka1-green fluorescent protein (GFP) and MoPax1-3×FLAG fusion constructs and cotransformed them into the Ku80 strain. The resulting transformants were screened by PCR, and their total proteins were examined by Western blotting analysis with anti-GFP and anti-FLAG antibodies. Total proteins isolated from the transformants were then incubated with anti-GFP beads. Proteins bound to the anti-GFP beads were eluted and subjected to Western blot analysis. The MoPax1 band was largely detected with the anti-FLAG antibody in the proteins eluted from the anti-GFP beads of the transformants coexpressing the MoMka1-GFP and MoPax1-3×FLAG fusion proteins (Fig. 1B). The results of the coimmunoprecipitation (co-IP) assays further confirmed that MoMka1 physically interacted with MoPax1 in M. oryzae.

Bioinformatics analysis displayed that MoMka1 was an ortholog of Bem1 in Saccharomyces cerevisiae (amino acid identity of 26.46%). Moreover, MoMka1 possesses two SH3 domains at the N terminus and PX and PB1 domains at the C terminus (Fig. 1C). To investigate the roles of these domains of MoMka1 in the interaction with MoPax1, we generated four MoMka1 variants (Fig. 1C). Pairs comprising different combinations of the truncated constructs of MoMka1 with MoPax1 were cotransformed into S. cerevisiae strain Y2H Gold. Y2H assays revealed that the second SH3 domain of MoMka1 is required for the interaction with MoPax1 (Fig. 1D), indicating that MoPax1 binds to the second SH3 domain of MoMka1.

### MoMka1 is involved in aerial hyphal growth, conidiogenesis, and conidial morphology.

**FIG 2:**
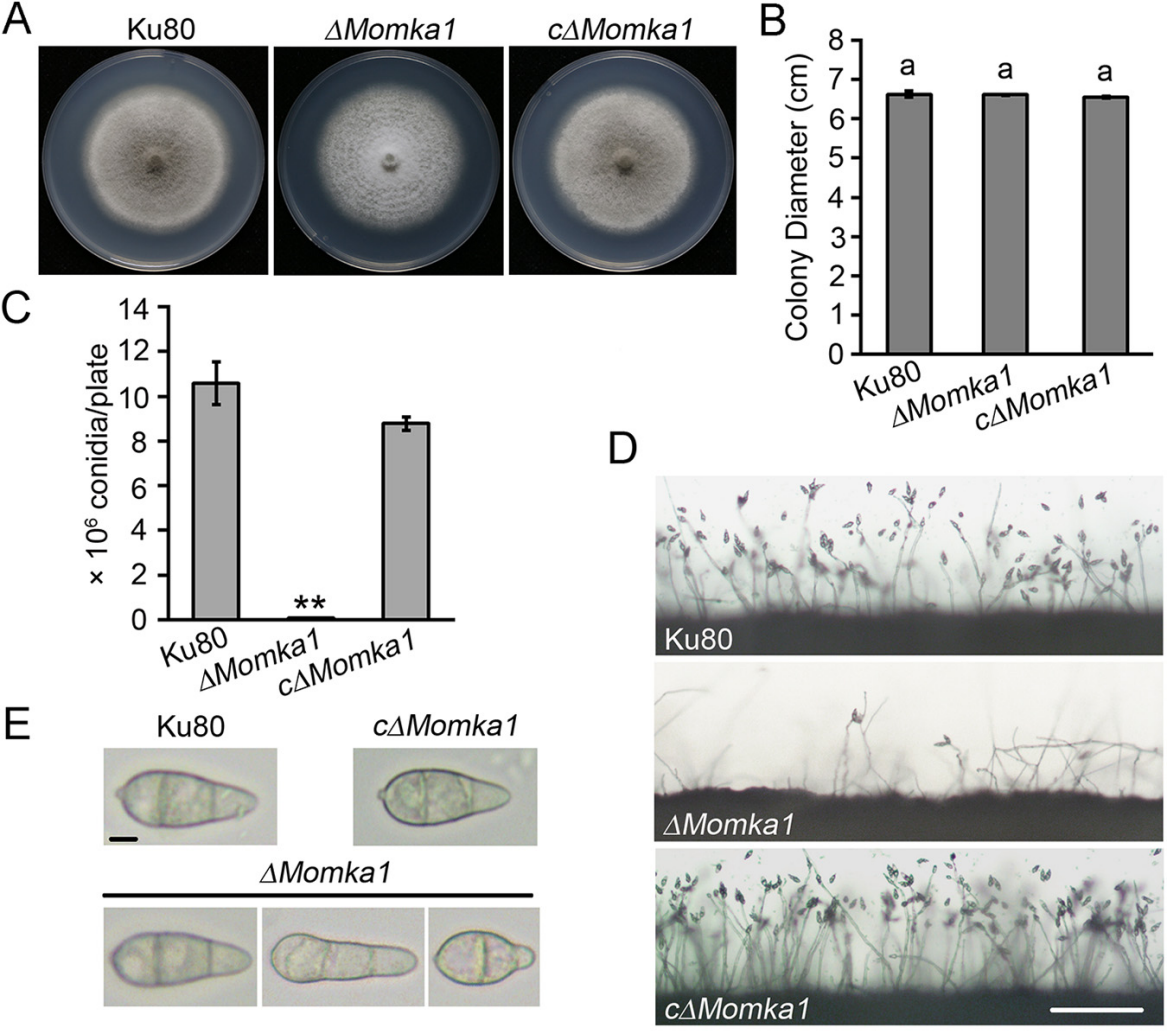
The phenotype analysis of the *ΔMomka1* mutant. (A) Colonies of the Ku80 strain, the *ΔMomka1* mutant, and the c*ΔMomka1* complemented strain cultured on CM plates at 25°C for 10 days. (B) Radial growth of each strain on CM plates. Error bars represent standard deviations. The same small letter indicates no significant difference (*P* < 0.05). (C) Bar chart showing statistical analysis of conidiation. Each strain was cultured on CM plates at 25°C for 12 days. Error bars represent standard deviations. **, *P* < 0.01. (D) Microscopic observation of conidial development. Conidial formation of each strain was observed under a light microscope after incubation on coverslips at 28°C for 24 h. Bar = 100 μm. (E) Microscopic observation of conidial morphology. Conidia of each strain were collected from the CM plates after growth at 25°C for 12 days. Bar = 10 μm.

To investigate the biological role of *MoMKA1* in M. oryzae, we generated targeted gene deletion mutants of *MoMKA1* via the homologous recombination strategy. The mutants (*ΔMomka1*) were confirmed by PCR and Southern blot analysis (Fig. S1 in the supplemental material). We subsequently obtained a complemented strain by reintroducing the *MoMKA1-GFP* gene fusion under the control of its native promoter into the *ΔMomka1* mutant. To determine the roles of *MoMKA1* in fungal morphogenesis, all tested strains were grown on CM plates (17). By 10 days, we observed that the *ΔMomka1* mutant formed colonies with significantly limited aerial hyphal growth compared with that of the Ku80 strain and the c*ΔMomka1* complemented strain (Fig. 2A), indicating that the deletion of *MoMKA1* results in much less aerial hyphal growth. However, the radial growth rates of all of the tested strains displayed no significant difference (Fig. 2B). The ability of the strains to produce conidia was evaluated by washing the surface of 10-day-old cultures. The *ΔMomka1* mutant produced significantly reduced conidia with (0.94 ± 0.15) × 10^6^ spores/plate (mean ± standard deviation), whereas the Ku80 strain produced (10.65 ± 0.67) × 10^6^ spores/plate (Fig. 2C and D). Interestingly, only 51.62% ± 2.61% conidia produced by the *ΔMomka1* mutant showed the normal pyriform morphology, while the rest of the conidia were abnormal, presenting elongated pyriform (37.99% ± 0.78%) or round (10.39% ± 2.08%) morphology (Fig. 2E). All of the phenotypical defects of the *ΔMomka1* mutant were restored in the c*ΔMomka1* complemented strain (Fig. 2A, C, D, and E and see Fig. 4). These results suggest that *MoMKA1* plays an important role in the aerial hyphal growth, conidiogenesis, and conidial morphology of M. oryzae.

10.1128/mbio.02218-22.1 FIG S1 Targeted gene replacement of *MoMKA1* and confirmation by PCR and Southern blot. (A) Construction of the vector pKOV21-MKA1 and targeted replacement of *MoMKA1*. B, BamHI; E, EcoRI. (B, C) PCR analysis for confirmation of the existence of the *MoMKA1* gene. (D) The expression levels of *MoMKA1* in the Ku80 strain, the *ΔMomka1* mutant, and the c*ΔMomka1* complemented strain were confirmed by qRT-PCR. (E) Southern blot analysis. Genomic DNA of each strain was digested with BamHI and EcoRI and hybridized with a ~1-kb probe amplified with the primers P-F/P-R. Download FIG S1, TIF file, 2.6 MB. Copyright © 2022 Lv et al. 2022 Lv et al. https://creativecommons.org/licenses/by/4.0/ This content is distributed under the terms of the Creative Commons Attribution 4.0 International license.

### *MoMKA1* is crucial for appressorium formation and appressorium-mediated plant infection by M. oryzae.

**FIG 3:**
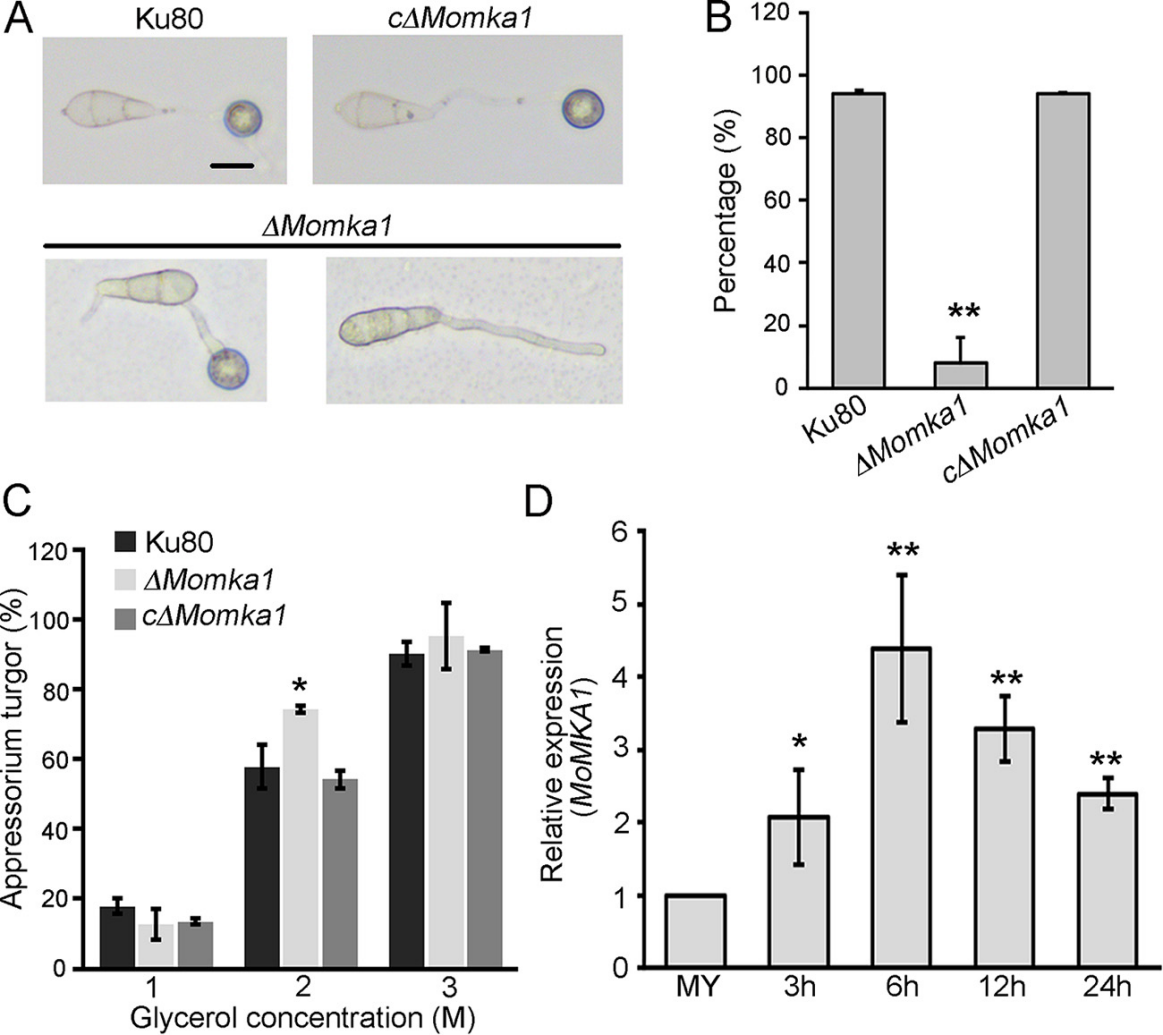
*MoMKA1* is involved in appressorium formation in M. oryzae. (A) Microscopic observation of appressorium development of the Ku80 strain, the *ΔMomka1* mutant, and the c*ΔMomka1* complemented strain. Conidia of each strain were harvested from the CM plates and allowed to form appressoria on hydrophobic surfaces (24 h). Bar = 10 μm. (B) Statistical analysis of appressorium formation rates. Error bars represent standard deviations. **, *P* < 0.01. (C) Quantification of collapsed appressoria of each strain. For each glycerol concentration, at least 100 appressoria were observed. Error bars represent standard deviations. *, *P* < 0.05. (D) The pattern of expression of *MoMKA1* during different stages of development by qRT-PCR analysis. MY, mycelium. *, *P* < 0.05; **, *P* < 0.01.

**FIG 4:**
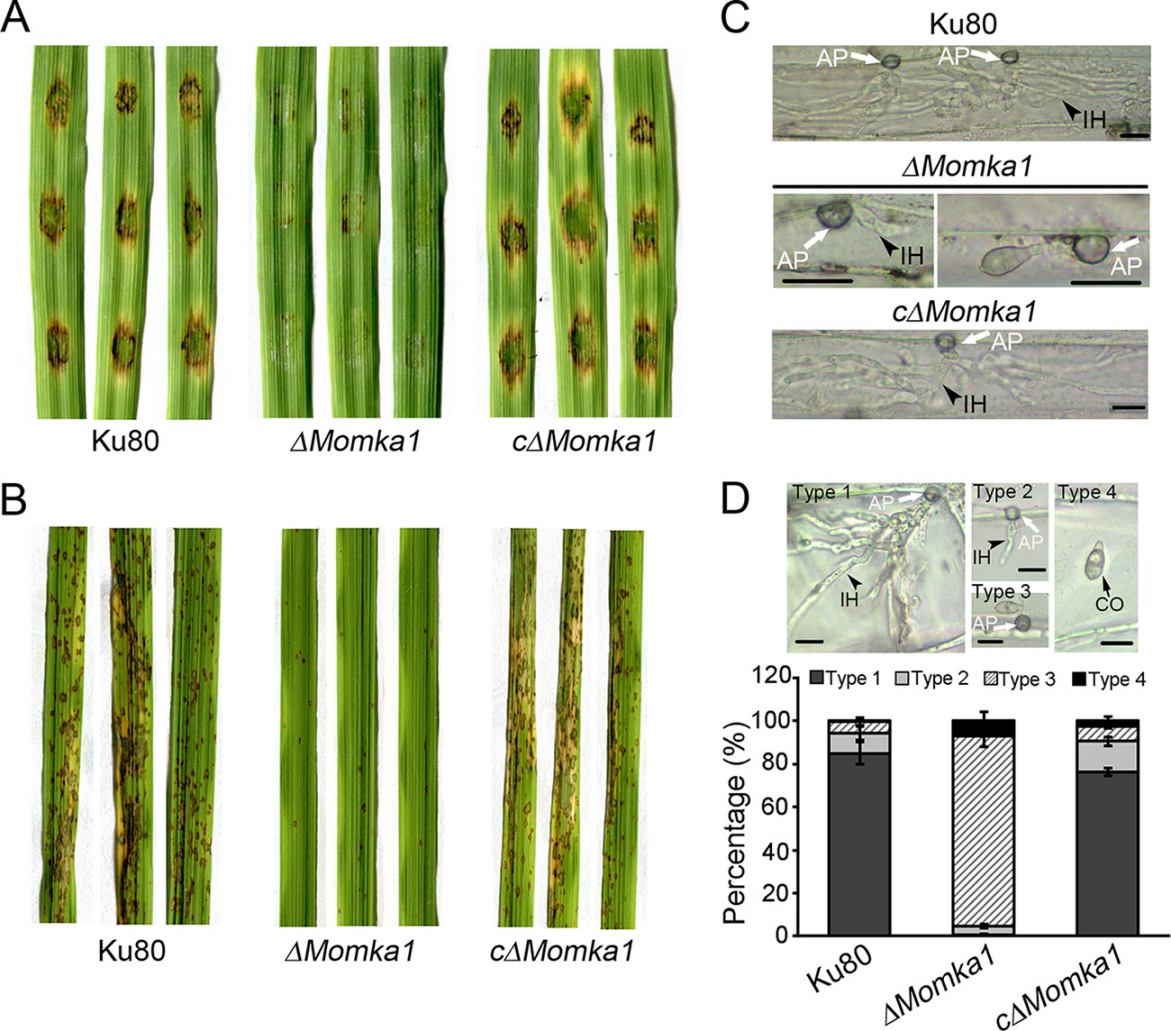
*MoMKA1* is essential for pathogenicity in M. oryzae. (A) Barley cut-leaf assays. With 20 μL per drop, conidial suspensions (5 × 10^4^ conidia/mL) of each strain were inoculated onto detached barley leaves. Photographs were taken at 5 dpi. (B) Spray inoculation assays. Rice seedlings were spray inoculated with 10 mL conidial suspension (2 × 10^4^ conidia/mL) of each strain. Photographs were taken at 5 dpi. (C) Penetration assays in the epidermis of barley leaves. Conidial suspensions (5 × 10^4^ conidia/mL) of each strain were drop inoculated onto the underside of detached barley leaves. After 24 h at 28°C in dark, the epidermis of barley leaves was torn and observed under a light microscope. AP, appressorium; IH, invasion hyphae. Bar = 20 μm. (D) Statistical analysis of penetration and invasion growth on onion epidermis cells. Error bars represent standard deviations. Type 1, invasion hyphae with branches; type 2, invasion hyphae with a single branch; type 3, no penetration; type 4, no germination. AP, appressorium; IH, invasion hyphae; CO, conidia. Bar = 20 μm.

To determine the role of MoMka1 in appressorium development, conidia of different strains were allowed to germinate on hydrophobic coverslips. By 24 h postinoculation (hpi), about 94% of conidia from the Ku80 strain and the c*ΔMomka1* complemented strain germinated and formed melanized appressoria at the tips of the germ tubes. However, only 8% of conidia from the *ΔMomka1* mutant produced appressoria on the hydrophobic surface (Fig. 3A and B), indicating that *MoMKA1* is involved in appressorium formation in M. oryzae. In 2 M glycerol, 57.74% ± 4.49% of Ku80 strain appressoria and 54.19% ± 9.42% of c*ΔMomka1* complemented strain appressoria had collapsed, compared to 74.34% ± 1.02% of appressoria of the *ΔMomka1* mutant (Fig. 3C). This result showed that appressorium turgor was reduced significantly in the *ΔMomka1* mutant. Besides this, during appressorium development, the expression level of the *MoMKA1* gene was significantly upregulated compared to its expression in the Ku80 strain (Fig. 3D). We further examined the pathogenicity of the *ΔMomka1* mutant by inoculating susceptible isolated intact barley leaves or abraded barley leaves with conidial suspensions. By 5 days postinoculation (dpi), large chlorotic blast lesions were observed at the inoculation sites of unwounded and wounded barley leaves inoculated with the Ku80 strain or the c*ΔMomka1* complemented strain, while only slightly dark or small brown spots on inoculated leaves were exhibited for inoculation with the *ΔMomka1* mutant (Fig. 4A; Fig. S2). Furthermore, the pathogenicity of the *ΔMomka1* mutant was tested by spray inoculating the seedlings of susceptible rice variety Co39 with conidial suspensions. By 5 dpi, the Ku80 and c*ΔMomka1* strains caused numerous typical blast lesions (gray spots with brown margins). In contrast, very few tiny lesions were produced by treatment with the *ΔMomka1* mutant (Fig. 4B). These results indicate that *MoMKA1* is crucial for plant infection by M. oryzae.

10.1128/mbio.02218-22.2 FIG S2 Pathogenicity assays on wounded barley cut leaves. With 20 μL per drop, conidial suspensions (5 × 10^4^ conidia/mL) of each strain were inoculated onto the wounded barley leaves. Photographs were taken at 5 dpi. Download FIG S2, TIF file, 2.2 MB. Copyright © 2022 Lv et al. 2022 Lv et al. https://creativecommons.org/licenses/by/4.0/ This content is distributed under the terms of the Creative Commons Attribution 4.0 International license.

To understand the reasons for the reduced pathogenicity of the *ΔMomka1* mutant, we examined the development of the infection structure by performing barley epidermis penetration assays. By 48 hpi, most conidia of Ku80 and the c*ΔMomka1* complemented strain germinated and formed appressoria, and dense infectious hyphae were observed in barley epidermis cells. However, most appressoria formed by the *ΔMomka1* mutant only produced penetration pegs or short unbranched invasive hyphae and were unable to grow or extend in cells (Fig. 4C). To quantitatively evaluate fungal infectious development on onion epidermis, the infection structures were divided into four types (Fig. 4D). Then, these different infection structures of each tested strain were counted. The results showed that more than 75% of conidia of the Ku80 and c*ΔMomka1* strains produced type 1 infectious hyphae. In contrast, about 90% of conidia of the *ΔMomka1* mutant did not penetrate the onion epidermis (Fig. 4D). Even when the *ΔMomka1* mutant occasionally penetrated into epidermis cells, the invasive growth was extremely impaired (Fig. 4C and D). These results suggest that *MoMKA1* is crucial for penetration and infectious growth in M. oryzae.

### MoMka1-GFP mainly distributes in the cytoplasm but also localizes to conidium septum holes, tip, and appressorium ring.

**FIG 5:**
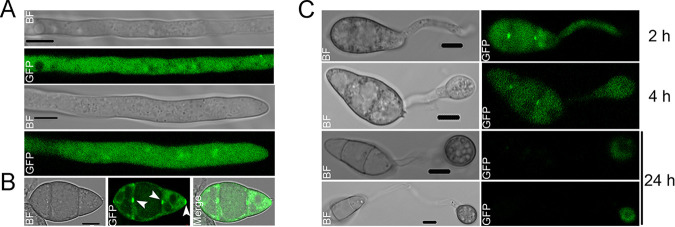
Subcellular localization of MoMka1 in hyphae (A), conidia (B), and mature appressoria (C) of the c*ΔMomka1* complemented strain. GFP fluorescence was examined under fluorescence confocal microscopy. Arrowheads point to punctate green fluorescence. BF, bright-field microscopy. Bar = 5 μm.

To understand the subcellular localization of MoMka1, GFP fluorescence was examined in the mycelium, conidia, and appressoria of the c*ΔMomka1* strain. We found that MoMka1-GFP mainly distributed throughout the cytoplasm in hyphae and conidia of the c*ΔMomka1* complemented strain (Fig. 5A). However, we also observed punctate green fluorescence around septum holes and at the tip of a conidium of the c*ΔMomka1* complemented strain (Fig. 5B). In mature appressoria of the c*ΔMomka1* complemented strain, we observed a GFP fluorescence ring (Fig. 5C). It has been reported that MoSec15, a component of the exocyst complex, localizes specifically in a ring formation at the appressorium pore in M. oryzae (20). To determine whether MoMka1 is a potential component of the ring, we carried out Y2H assays. The Y2H analysis showed that MoMka1 directly interacts with MoSec15 (Fig. S3), indicating that the localization of MoMka1 to the appressorium ring is mediated by MoSec15 in M. oryzae.

10.1128/mbio.02218-22.3 FIG S3 MoMka1 interacts with MoSec15. (A) The fusion constructs *MoMKA1*-pGADT7 and *MoSEC15*-pGBKT7 were cointroduced into the yeast Y2H Gold strain, and transformants were cultured on SD-Leu-Trp plates as a control and on SD-Ade-Leu-Trp-His for 3 days. The pair of plasmids pGADT7-T and pGBKT7-53 was used as the positive control, and the pair of plasmids pGADT7-T and pGBKT7-Lam was used as the negative control. (B) Co-IP assays for the interaction between MoMka1 and MoSec15. The MoMka1-GFP and MoSec15-3×FLAG fusion constructs were coexpressed in the Ku80 strain. The isolated proteins were analyzed by Western blotting using anti-GFP and anti-Flag antibodies. Download FIG S3, TIF file, 0.8 MB. Copyright © 2022 Lv et al. 2022 Lv et al. https://creativecommons.org/licenses/by/4.0/ This content is distributed under the terms of the Creative Commons Attribution 4.0 International license.

### MoMka1 interacts with Mst50.

**FIG 6:**
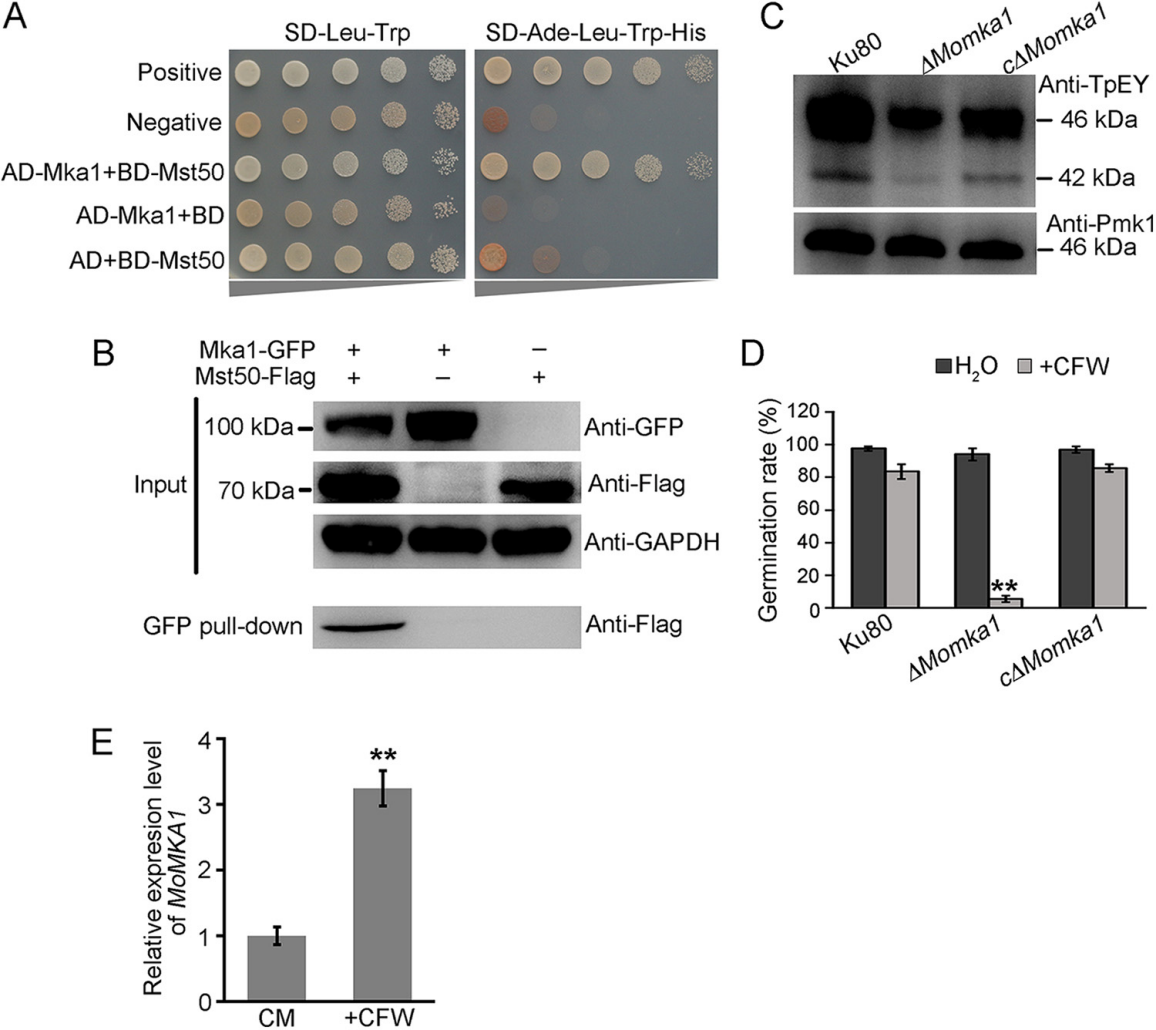
MoMka1 interacts with Mst50 and is involved in MAPK signaling pathways in M. oryzae. (A) Y2H analysis of the interaction between MoMka1 and Mst50. The fusion constructs *MoMKA1*-pGADT7 and *MST50*-pGBKT7 were cointroduced into the yeast Y2H Gold strain, and transformants were cultured on SD-Leu-Trp plates as a control and on SD-Ade-Leu-Trp-His for 3 days. The pair of plasmids pGADT7-T and pGBKT7-53 was used as the positive control, and the pair of plasmids pGADT7-T and pGBKT7-Lam was used as the negative control. (B) Co-IP assays for the interaction between MoMka1 and Mst50. Total proteins (input) extracted from the strains containing the Mst50-3×FLAG and MoMka1-GFP constructs or a single construct (Mst50-3×FLAG or MoMka1-GFP) were subjected to SDS-PAGE, and immunoblots were incubated with anti-FLAG and anti-GFP antibodies. In addition, each protein sample was pulled down using anti-FLAG antibody and further detected with anti-GFP antibody (IP). The protein samples were also detected with anti-GAPDH antibody as a reference. (C) TpEY phosphorylation assays. Total proteins were isolated from mycelia of each strain. The anti-Pmk1 antiserum detected a 42-kDa Pmk1 band in all the samples. An anti-TpEY antibody detected the phosphorylation levels of Pmk1 and Mps1 (46 kDa). (D) Conidial germination assays. Conidial suspensions of each strain were inoculated onto hydrophobic surfaces with or without 50 μg/mL calcofluor white (CFW). Mean values and standard deviations of the germination rates assayed at 6 h were calculated from three replicates. Error bars represent standard deviations. **, *P* < 0.01. (E) The relative expression levels of *MoMKA1* in CM liquid medium with or without CFW by qRT-PCR analysis. Error bars represent standard deviations. **, *P* < 0.01.

MoMka1 was predicted as an MAP kinase activator and, as in mammals, it probably acts upstream from the MAPK cascades. Thus, we sought to identify the interactions between MoMka1 and MAPK-Pmk1 components by performing the Y2H assays. The results showed that MoMka1 directly interacts with Mst50 (Fig. 6A), but not Mst11, Mst7, and Pmk1 (Fig. S4A). To further verify the interaction between MoMka1 and Mst50, we carried out co-IP assays. MoMka1-GFP and Mst50-3×FLAG fusion constructs were generated and cotransformed into the Ku80 strain. The resulting transformants were screened by PCR and further examined by Western blotting analysis with the anti-GFP and anti-FLAG antibodies (Fig. 6B). Total proteins isolated from the transformants were then incubated with anti-GFP beads. Proteins bound to anti-GFP beads were eluted and subjected to Western blot analysis. The Mst50 band was largely detected with the anti-FLAG antibody in the proteins eluted from anti-GFP beads of the transformants coexpressing the MoMka1-GFP and Mst50-3×FLAG fusion proteins (Fig. 6B). The results of co-IP assays further confirmed that MoMka1 physically interacts with Mst50 in M. oryzae.

10.1128/mbio.02218-22.4 FIG S4 The Y2H assays. (A) Negative interactions between MoMka1 and Mst11, Mst7, Pmk1, MoMsb2, MoCdc42, MoCDC24, and MoNoxR. All transformants were cultured on SD-Ade-Leu-Trp-His for 3 days. (B) MoMka1 interacts with MoSte5. The fusion constructs *MoMKA1*-pGADT7 and *MoSTE5*-pGBKT7 were cointroduced into the yeast Y2H Gold strain, and transformants were cultured on SD-Leu-Trp plates as a control and on SD-Ade-Leu-Trp-His for 3 days. The pair of plasmids pGADT7-T and pGBKT7-53 was used as the positive control, and the pair of plasmids pGADT7-T and pGBKT7-Lam was used as the negative control. (C) MoMka1 interacts with Mst20. The fusion constructs *MoMKA1*-pGADT7 and *MST20*-pGBKT7 were cointroduced into the yeast Y2H Gold strain, and transformants were cultured on SD-Leu-Trp plates as a control and on SD-Ade-Leu-Trp-His for 3 days. Download FIG S4, TIF file, 2.2 MB. Copyright © 2022 Lv et al. 2022 Lv et al. https://creativecommons.org/licenses/by/4.0/ This content is distributed under the terms of the Creative Commons Attribution 4.0 International license.

### MoMka1 functions upstream from the Pmk1 and Mps1 MAPK pathways.

To determine the role of MoMka1 in the activation of Pmk1 and Mps1 MAPK pathways, we carried out Western blotting with an anti-TpEY antibody. In proteins isolated from vegetative hyphae harvested from 2-day-old liquid CM cultures, no significant difference in the phosphorylation of either Pmk1 or Mps1 was detected among the Ku80 strain, the *ΔMomka1* mutant, and the c*ΔMomka1* complemented mutant (data not shown). Previously, it has been reported that the phosphorylation level of Pmk1 was significantly higher in aerial hyphae of the *MoRAS2^G18V^* strain than in those of the Ku80 strain, but not in vegetative hyphae from liquid CM cultures (21). Therefore, we collected aerial hyphae of each tested strain from 2-week-old CM plates and subjected them to immunoblotting with the anti-TpEY antibody. In proteins isolated from aerial hyphae, the phosphorylation levels of Pmk1 and Mps1 were significantly reduced in the *ΔMomka1* mutant compared to their phosphorylation levels in the Ku80 and c*ΔMomka1* strains (Fig. 6C), suggesting that MoMka1 is involved in Pmk1 and Mps1 phosphorylation.

Mps1 is required for the regulation of cell wall integrity in M. oryzae (9). Since the phosphorylation level of Mps1 was reduced in the *ΔMomka1* mutant, we assayed the cell wall integrity defects of the *ΔMomka1* mutant. In the presence of 50 μg/mL calcofluor white (CFW), more than 83% of conidia produced by the Ku80 and c*ΔMomka1* strains germinated after incubation in the dark at 28°C for 6 h. Under the same conditions, only approximately 6% of the *ΔMomka1* mutant conidia produced germ tubes (Fig. 6D), indicating that deletion of *MoMKA1* increases sensitivity to cell wall stress. Under the condition of no added CFW, conidia of the *ΔMomka1* mutant germinated as efficiently as those of the Ku80 and c*ΔMomka1* strains. Besides this, the expression of *MoMKA1* was significantly upregulated under 50 μg/mL CFW treatment (Fig. 6E).

Taken together, we conclude that MoMka1 functions upstream from the Pmk1 and Mps1 MAPK pathways in M. oryzae.

### MoMka1 interacts with MoAtg13 and MoAtg6.

**FIG 7:**
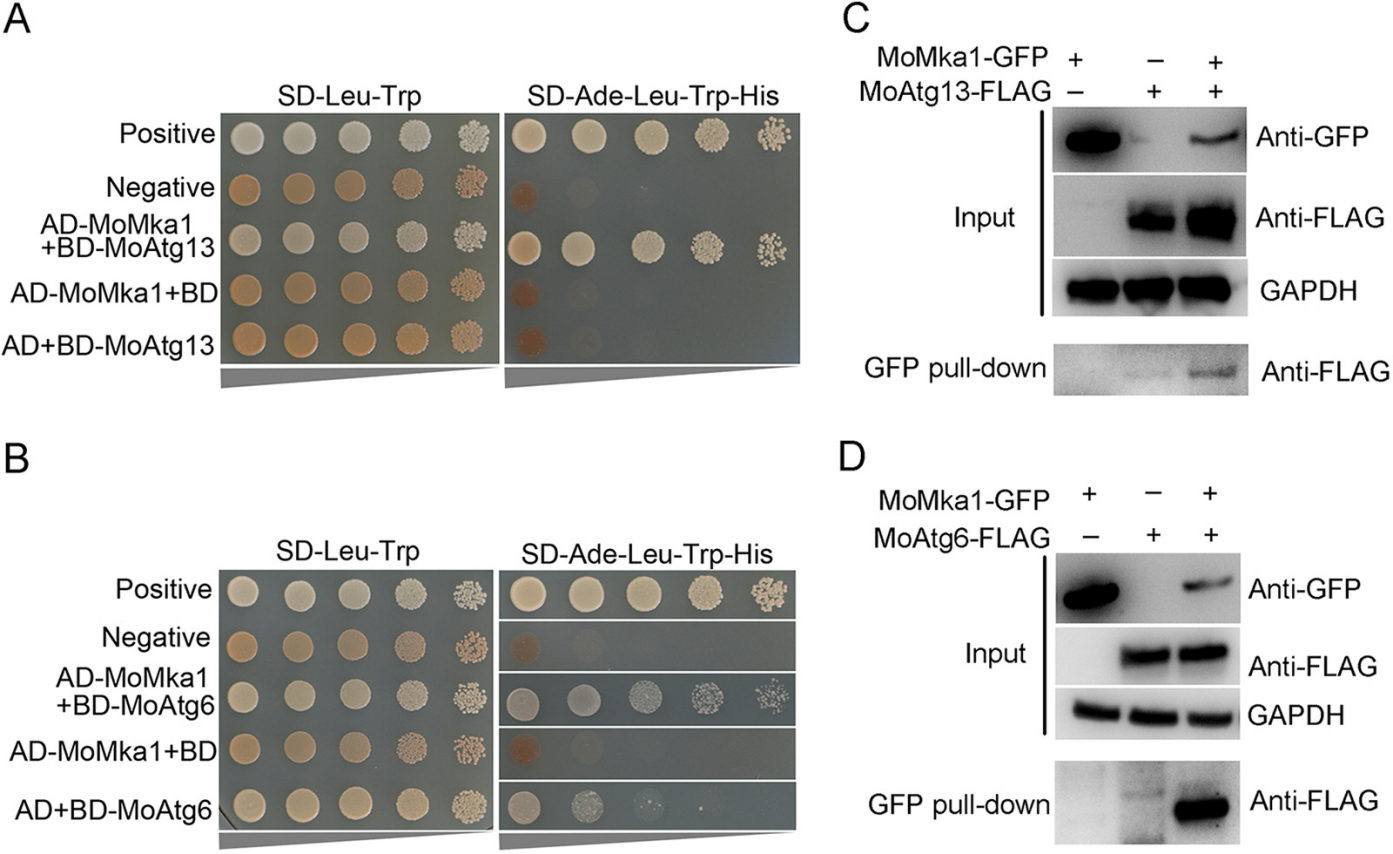
MoMka1 interacts with MoAtg13 and MoAtg6. (A) The Y2H assay of interaction between MoMka1 and MoAtg13. Serial concentrations of yeast cells transformed with bait and prey constructs indicated in the figure were assayed for growth on SD-Ade-Leu-Trp-His for 3 days. (B) Y2H analysis of the interaction between MoMka1 and MoAtg6. (C) Co-IP assays for the interaction between MoMka1 and MoAtg13. Total proteins (input) extracted from the strains containing the MoMka1-GFP and MoAtg13-3×FLAG constructs or a single construct (MoMka1-GFP or MoAtg13-3×FLAG) were probed with anti-GFP and anti-FLAG antibodies. In addition, each protein sample was pulled down using anti-FLAG antibody and further detected with anti-GFP antibody (IP). The protein samples were also detected with anti-GAPDH antibody as a reference. (D) Co-IP assays for the interaction between MoMka1 and MoAtg6.

In mammals, autophagy can be induced in response to loss of integrin-mediated attachment, and autophagy promotes focal adhesion disassembly and cell motility through the direct interaction of paxillin with LC3 (22–24). In addition, MAPK signaling pathways have been implicated in autophagy regulation (25, 26). Therefore, we analyzed the interaction between MoMka1 and Atg candidates (such as MoAtg1, MoAtg6, MoAtg13, and MoAtg18) with Y2H assays. We found that MoMka1 interacted with both MoAtg13 and MoAtg6 (Fig. 7A and B). To further validate the interactions between MoMka1 and MoAtg13 (or MoAtg6), we carried out co-IP assays. MoMka1-GFP and MoAtg13-3×FLAG (or MoAtg6-3×FLAG) fusion constructs were generated and transformed into Ku80. The resulting transformants were screened by PCR and further examined by Western blotting analysis with the anti-GFP and anti-FLAG antibodies (Fig. 7C and D). The MoAtg13 or MoAtg6 band was largely detected with the anti-FLAG antibody in proteins eluted from anti-GFP beads of the transformants coexpressing the MoMka1-GFP and MoAtg13-3×FLAG or MoAtg6-3×FLAG fusion proteins (Fig. 7C and D). These results of co-IP assays further confirmed that MoMka1 interacts with both MoAtg13 and MoAtg6 in M. oryzae.

### MoMka1 regulates autophagy in M. oryzae.

**FIG 8:**
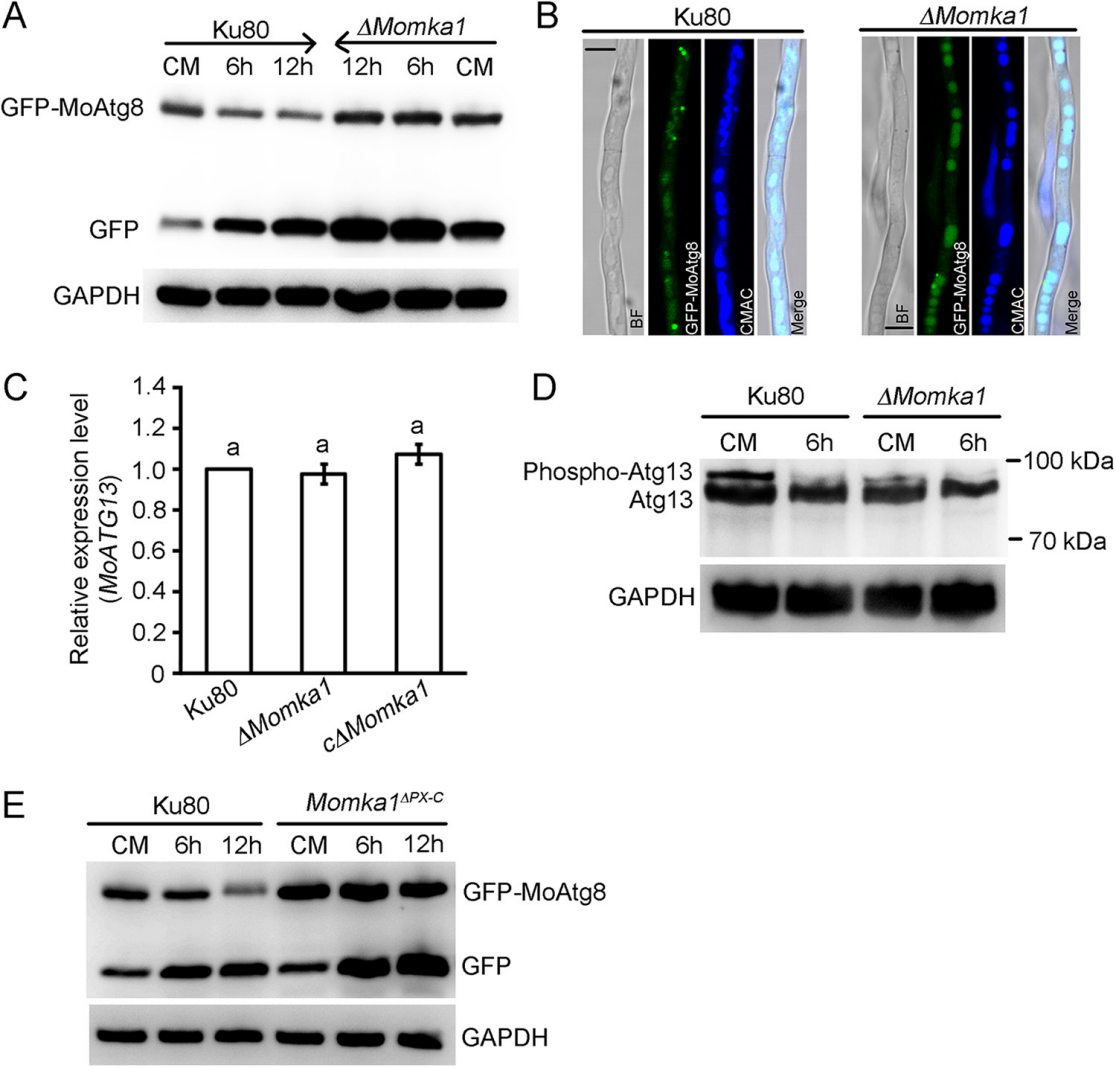
*MoMKA1* regulates autophagy through affecting the phosphorylation level of MoAtg13 in M. oryzae. (A) GFP-MoAtg8 proteolysis assays of the Ku80 strain and the *ΔMomka1* mutant expressing the GFP-MoAtg8 fusion construct. Total proteins were extracted for analysis by Western blotting with anti-GFP antibody. Anti-GAPDH antibody was used as a control. (B) GFP-MoAtg8 localization in the hyphal cells of Ku80 and the *ΔMomka1* mutant under nutrient-rich conditions (CM medium). The vacuoles were stained with CMAC (7-amino-4-chloromethycoumarin) and examined by fluorescence microscopy. Bar = 5 μm. (C) The expression level of *MoATG13* in each strain cultured in CM medium as determined by qRT-PCR analysis. Error bars represent standard deviations. The same small letter indicates no significant difference (*P* < 0.05). (D) Detection of the MoAtg13 phosphorylation level in Ku80 and the *ΔMomka1* mutant. Lysates derived from Ku80 and the *ΔMomka1* mutant cultured in CM liquid medium and MM-N medium for 6 h were subjected to SDS-PAGE and immunoblotting with anti-Atg13 antibody. Anti-GAPDH antibody was also used as a control. (E) GFP-MoAtg8 proteolysis assays of Ku80 and the *Momka1^ΔPX-C^* mutant with the PX domain deleted. Total proteins were extracted for analysis by Western blotting with anti-GFP antibody. Anti-GAPDH antibody was used as a control.

Since MoMka1 interacts with the key autophagy components MoAtg13 and MoAtg6, we decided to investigate potential roles of MoMka1 in regulating autophagy. We introduced an autophagy marker fusion construct, GFP-MoAtg8, into the Ku80 strain and the *ΔMomka1* mutant. Autophagy flux was analyzed by the GFP-MoAtg8 proteolysis. In both Ku80 and the *ΔMomka1* mutant expressing *GFP-MoATG8*, a full-length GFP-MoAtg8 fusion protein (42 kDa) and free GFP (27 kDa) could be readily detected in immunoblots with an anti-GFP antibody. When cultured in liquid CM medium, the Ku80 strain contained smaller amounts of free GFP than of GFP-MoAtg8 (Fig. 8A), suggesting relatively low levels of autophagic flux. In contrast, the *ΔMomka1* mutant accumulated larger amounts of free GFP than of GFP-MoAtg8 in CM medium (Fig. 8A), indicating that increased autophagic flux is a consequence of *MoMKA1* deletion. Upon nitrogen starvation (MM-N liquid medium), free GFP became increasingly abundant in both the Ku80 strain and the *ΔMomka1* mutant as the induction time was extended, but the autophagic fluxes in the two strains showed no significant difference. These results indicate that the autophagic flux is stronger in the *ΔMomka1* mutant than in Ku80 under nutrient-rich but not under starvation conditions.

We further assessed autophagic flux under nutrient-rich conditions by epifluorescence microscopy. When grown in CM, GFP-MoAtg8 was observed in hyphae as punctate structures and lower levels of GFP-MoAtg8 fluorescence accumulated in vacuoles in the Ku80 strain. In contrast, most GFP-MoAtg8 accumulated inside vacuoles and fewer punctate structures of GFP-MoAtg8 fluorescence were displayed in hyphae of the *ΔMomka1* mutant (Fig. 8B). The results indicate that vacuolar delivery and fusion of autophagosomes with the vacuole are increased in the *ΔMomka1* mutant under nutrient-rich conditions. We conclude that MoMka1 plays an important role in maintaining homeostatic regulation of autophagy in M. oryzae.

In S. cerevisiae, Atg13 forms the Atg1 complex with Atg1 and the Atg17-Atg31-Atg29 subcomplex and is essential for autophagy initiation and autophagosome formation (27, 28). Under nutrient-rich conditions, Atg13 is hyperphosphorylated by target of rapamycin complex 1 (TORC1) and then inhibits the formation of the Atg1 complex. Upon inactivation of TOR, Atg13 is rapidly dephosphorylated, allowing the Atg1 complex to assemble and induce autophagy (29). Due to the interaction between MoMka1 and MoAtg13, we hypothesized that MoMka1 regulates autophagy by modulating the phosphorylation level of MoAtg13. To test this hypothesis, we first measured the expression level of *MoATG13* in each strain. The expression level of *MoATG13* displayed no significant difference in the *ΔMomka1* mutant compared to the levels in the Ku80 strain and the c*ΔMomka1* complemented strain (Fig. 8C). By further examining the MoAtg13 contents with anti-Atg13 antibody, we found that the levels of MoAtg13 displayed no significant difference between the Ku80 strain and the *ΔMomka1* mutant under both the nutrient-rich condition and the nitrogen-starvation condition (Fig. 8D). The phosphorylation levels of MoAtg13 were similar in both the Ku80 strain and the *ΔMomka1* mutant under the nitrogen-starvation condition (Fig. 8D). However, the phospho-MoAtg13 band of the *ΔMomka1* mutant was significantly weaker than that of the Ku80 strain under the nutrient-rich condition (Fig. 8D). The results suggest that deletion of *MoMKA1* leads to inhibition of MoAtg13 phosphorylation and cell autophagy under nutrient-rich conditions in M. oryzae.

Yeast Atg13 contains a HORMA domain at the N terminus that is required for recruitment of the phosphatidylinositol (Ptdlns) 3-kinase subunit Atg14 (27, 30). Moreover, it has been reported that MoAtg14 interacts with MoAtg6 in M. oryzae (31). Since MoMka1 interacts with MoAtg13 (or MoAtg6) (Fig. 1) and the MoMka1 PX domain binds Ptdlns 3-phosphate (PI3P) (32), we speculated that MoMka1 might bind PI3P to regulate cell autophagy. To verify the hypothesis, we deleted the PX domain in the *MoMKA1* gene to generate the *Momka1^ΔPX-C^* mutant and then introduced the fusion construct GFP-MoAtg8 into the *Momka1^ΔPX-C^* mutant for assessing autophagy flux by analyzing the GFP-MoAtg8 proteolysis. When cultured in liquid CM medium, both the Ku80 strain and the *Momka1^ΔPX-C^* mutant contained smaller amounts of free GFP than of GFP-MoAtg8 (Fig. 8E), suggesting relatively low levels of autophagic flux. Upon nitrogen starvation, free GFP became increasingly abundant in both the Ku80 strain and the *Momka1^ΔPX-C^* mutant as the induction time was extended (Fig. 8E). These results indicated that the mechanism by which MoMka1 regulates autophagy is possibly not by the PX domain in M. oryzae.

## DISCUSSION

It has been well known that mammal cells utilize focal adhesions (FAs) to sense their environment and regulate mechanotransduction coupling with the extracellular matrix (ECM) in physiological processes like migration, growth, differentiation, and proliferation (33–35). FAs form on the cytoplasmic side of the plasma membrane as integrin receptors attach outside the cell to the ECM (36). Integrins are cell surface receptors transducing extracellular signals into cells from the ECM through interaction with the cytoskeleton, signaling molecules, and other cellular proteins, such as paxillin (37–39). Paxillin, as a multifunctional FA adaptor protein, can recruit several structural and signaling proteins, including vinculin, focal adhesion kinase (FAK), and Src (a proto-oncogenic tyrosine kinase) (18, 40). FAK, an important protein tyrosine kinase, mediates several integrin signaling pathways (41). FAK Tyr397 phosphorylation promotes Src binding, which leads to the activation of Src. Then, activated Src can also phosphorylate FAK at Tyr925, which creates an SH2-binding site for the adaptor protein growth factor receptor-bound protein 2 (Grb2) (42). Grb2 binds with SOS (Son of Sevenless), an important Ras guanine nucleotide exchange factor (GEF) that activates the membrane-anchored Ras GTPase and then activates the mitogen-activated protein kinase (MAPK) pathways (43). However, unlike in mammals, research on the integrin-FAK-MAPK signaling pathway is very limited in fungi. In Candida albicans, it has been revealed that an FAK-like protein, CaFak, is involved in the control of yeast cell adhesion to endothelial cells (44). Similarly, in Fusarium solani, it has been reported that the integrin-FAK signaling pathway is involved in the control of F. solani adhesion to human corneal epithelial cells (HCECs) (45). Unfortunately, in plant-pathogenic fungi, since the key components, such as integrin, FAK, and Src, are unable to be identified by BLAST analysis, the integrin-FAK-MAPK signaling pathway has not been characterized. In this study, we found a novel signaling regulatory mechanism by which MoPax1 activates MAP kinase signaling pathways and autophagy through MoMka1 during appressorium-mediated plant infection by M. oryzae. Obviously, the signaling networks described in this study are different from the conventional integrin-paxillin-FAK signaling pathway in mammal cells.

In M. oryzae, the Pmk1 MAPK pathway regulates appressorium formation and infectious hyphal growth and the Mps1 MAPK pathway modulates the fungal cell wall integrity to regulate appressorium penetration of the plant surface (7, 9). Mst50 serves as an adaptor of the Mst11-Mst7-Pmk1 and Mck1-Mkk2-Mps1 cascades, several interacting proteins that play important roles in fungal development and pathogenicity have been identified (10, 11). In this study, we identified a novel Mst50-interacting protein, MoMka1, that is crucial for appressorium-mediated plant infection. We found that only approximately 8% of conidia of the *ΔMomka1* mutant produced appressoria on a hydrophobic surface (Fig. 3A and B). Consistently, the expression level of the *MoMKA1* gene was significantly upregulated during the process of appressorium development (Fig. 3C). Moreover, the phosphorylation levels of Pmk1 and Mps1 in aerial hyphae of the *ΔMomka1* mutants were significantly reduced (Fig. 6C). When the conidia were treated with the cell wall integrity stress factor CFW, the germination of conidia from the *ΔMomka1* mutant was significantly inhibited (Fig. 6D). These results suggest that MoMka1 acts from upstream of the Pmk1 and Mps1 MAPK pathways during appressorium-mediated plant infection in M. oryzae.

In this study, one of the most interesting findings is that MoMka1 interacts with autophagy-related proteins (MoAtg13 and MoAtg6) and is involved in the autophagy process in M. oryzae. Autophagy is a conserved cellular degradation process that functions as a cellular homeostatic mechanism. In mammals, mounting evidence has demonstrated that autophagy is induced upon loss of integrin-mediated cell attachments to the surrounding ECM and is required for FA disassembly (23, 24). Paxillin colocalizes with autophagosomes and interacts directly with LC3 (an ortholog of yeast Atg8) in mammals (22). In a previous report, it was shown that Atg13 runs as a doublet in SDS-PAGE gels and loses the upper band upon autophagy stimulation (46). Similarly, in this study, two bands of MoAtg13 were also detected in M. oryzae by Western blotting using anti-Atg13 antibody. Under nutrient-rich conditions, phosphorylated MoAtg13 was significantly decreased in the *ΔMomka1* mutant (Fig. 8D), which suggests that *MoMKA1* regulates autophagy by affecting the phosphorylation level of MoAtg13 in M. oryzae. We provide the first evidence that MoMka1 plays a critical role in autophagy in M. oryzae.

The target of rapamycin (Tor) family of serine/threonine protein kinases inhibits the activation of autophagy by regulating the formation of the Atg1-Atg13-Atg17 complex through hyperphosphorylation of Atg13 (29). Tor directly phosphorylates Atg13 at multiple Ser residues (47, 48). Protein kinase A (PKA) also phosphorylates Atg13 at three conserved sites (S344, S437, and S581) (49). Besides this, Atg13 S224 is modulated by the AMP-activated protein kinase (AMPK) pathway (29), and phosphorylation of some residues (such as S318 and S355) depends on ULK1 (50). In this study, we found that the phosphorylation level of MoAtg13 declined in the *ΔMomka1* mutant under nutrient-rich conditions (Fig. 8D). However, the specific sites in MoAtg13 responsible for the decreased phosphorylation have not been determined. Previously, the MAPK signaling pathways have been implicated in autophagy regulation (17, 25, 26). Therefore, we speculate that the MAPK signaling pathways may be involved in the regulation of MoAtg13 phosphorylation and autophagy in M. oryzae.

MoMka1 is an ortholog of S. cerevisiae Bem1 according to BLASTP analysis. The Bem1 protein, containing SH3 domains, stimulates Fus3 kinase (MAPK) activity and associates with Ste5, the tethering protein of the Ste11-Ste7-Fus3 cascade pathway (51). Besides this, Bem1 acts as a scaffold protein critical for cell polarity and assists in the clustering of cell polarity proteins around sites of growth (52, 53). Bem1 in the dimorphic yeast Yarrowia lipolytica (YlBem1) was localized in vesicular structures dispersed throughout the cytosol and particularly concentrated at sites of growth, and it assembled at the mother-bud neck in the late stages of cell division (54). In the gray mold fungus Botrytis cinerea, the scaffold protein BcBem1 localizes in the cytoplasm and the septa and, rarely, at hyphal tips (55). In the other filamentous fungi, including Ustilago maydis, Aspergillus nidulans, and Neurospora crassa, the orthologs of Bem1 mainly localize at the cell tips (56–58). The above-described studies suggest a relatively conserved role of Bem1 orthologs in the regulation of fungal cell polarity. In this study, we observed that MoMka1-GFP localized in the cytoplasm of hyphal cells, but not at hyphal tips, and also accumulated at septum holes and the tips of conidial cells (Fig. 5), suggesting that MoMka1 may regulate cell polarity in conidia but not in hyphae.

Besides this, it has been reported that S. cerevisiae Bem1 interacts with the exocyst complex through subunit Sec15 at sites of growth (59). Also, in Candida albicans, Sec15 interacts directly with Bem1, and disruption of the interaction by shutting off *SEC15* results in mislocalization of Bem1-GFP (60). In M. oryzae, during plant infection-related development, the exocyst complex specially assembles in the appressorium at the point of plant infection (20). Consistently, we found by performing Y2H assays that MoMka1 interacts with MoSec15 in M. oryzae (Fig. S2), indicating that MoSec15 may play a critical role in mediating MoMka1’s localization to the appressorium ring. Moreover, it is possible that deletion of *MoMKA1* attenuates plant infection by disrupting appressorium repolarization. However, since the *MoSEC15* gene is essential in M. oryzae (20), we were unable to knock out *MoSEC15* to explore the influence of the cellular localization of MoMka1-GFP in a strain expressing *MoMKA1-GFP*. To further determine whether MoMka1 localizes to the appressorium ring, more experiments to display the colocalization of MoSec15 and MoMka1 are needed.

Bem1 binds to the cytoplasmic domain of transmembrane protein Msb2 and recruits two p21-activated kinases (PAKs), Ste20 and Cla4, to the membrane to activate the kinase Ste11 in S. cerevisiae (61). Previously, it has been reported that *MoMSB2*, an ortholog of yeast *MSB2*, functions upstream from the Pmk1 MAPK pathway in M. oryzae (62). Deletion of *MoMSB2* causes significantly reduced phosphorylation of Pmk1 (62). Therefore, we were interested in determining whether MoMsb2 interacted with MoMka1 in M. oryzae. However, unlike in yeast, the interaction between MoMka1 and MoMsb2 was negative in Y2H assays (Fig. S4A). In this study, we also determined several other interacting partners of MoMka1, including Mst20 and MoSte5 (Fig. S4B and C). Mst20, a member of the PAK family, has been reported to be required for aerial hyphal growth and conidiation but dispensable for appressorium formation, penetration, and plant infection in M. oryzae (63). MoSte5 is an ortholog of S. cerevisiae Ste5, a scaffold protein that promotes Fus3 activation (64). MoSte5 has not been functionally characterized yet. Therefore, it will be interesting to explore the biological roles of the MoMka1-MoSte5 complex in the future. In S. cerevisiae, Bem1 physically interacts with Cdc42, Cdc24, and NoxR at the cell tips (56, 65, 66). However, we did not detect direct interaction between MoMka1 and MoCdc42, MoCdc24, or MoNoxR in Y2H assays in M. oryzae (Fig. S4A).

**FIG 9:**
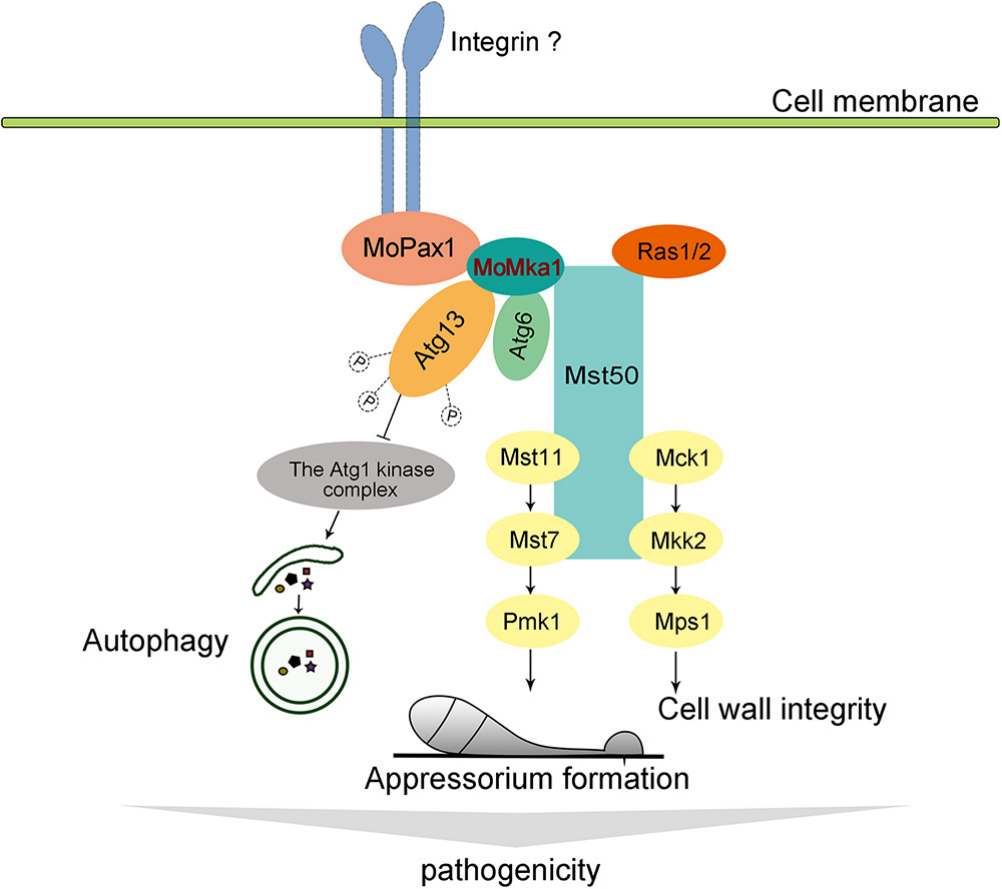
A model in which MoPax1 activates MAP kinase signaling pathways and autophagy through MoMka1 during appressorium-mediated plant infection by M. oryzae.

Based on previous work and this study, we describe a model wherein MoPax1 activates MAP kinase signaling pathways and autophagy through MoMka1 during appressorium-mediated plant infection by M. oryzae (Fig. 9). Various extracellular signals specifically recognize unknown transmembrane receptors, such as integrins, and the stimulation arising from the recognition may be transmitted to intracellular MoPax1. MAP kinase activator MoMka1, as an interaction protein of MoPax1, binds with Mst50 and regulates the phosphorylation levels of both Pmk1 and Mps1 to activate the MAPK signaling pathways, thereby regulating appressorium-mediated plant infection. Meanwhile, MoMka1 interacts with both MoAtg6 and MoAtg13 and controls autophagy initiation by influencing the phosphorylation level of MoAtg13. In summary, MoMka1 acts upstream from MAPK signaling pathways and the autophagy process to regulate morphogenesis and pathogenicity in M. oryzae.

## MATERIALS AND METHODS

### Strains, culture conditions, and phenotypic analyses.

The Ku80 strain of M. oryzae was used for generating the *ΔMomka1* mutant in this study. The growth and maintenance of M. oryzae and medium composition were described previously (67, 68). Escherichia coli strain 1-T1 was used for routine bacterial transformations and maintenance of plasmids in this study. The vegetative growth, conidiation ability, infection structure morphogenesis, and pathogenicity of M. oryzae were determined according to the methods described previously (67, 68).

### Generation of *MoMKA1* deletion mutants and complementation mutants.

The *MoMKA1* sequence was obtained from EnsemblFungi (http://fungi.ensembl.org/Magnaporthe_oryzae/Info/Index?db=core). The domains of MoMka1 were identified using SMART (http://smart.embl.de/smart/set_mode.cgi?NORMAL=1). To construct *MoMKA1* gene replacement vector pKO-MKA1, the *HPH* gene cassette was fused with the upstream and downstream flanking fragments, which were amplified with primer pairs UP-F/UP-R and Down-F/Down-R, respectively (Table S1). The gene replacement vector pKO-MKA1 was then transformed into protoplasts of the Ku80 strain to generate null mutants, as previously described (13).

10.1128/mbio.02218-22.5 TABLE S1 Primers used in this study. Download Table S1, DOCX file, 0.02 MB. Copyright © 2022 Lv et al. 2022 Lv et al. https://creativecommons.org/licenses/by/4.0/ This content is distributed under the terms of the Creative Commons Attribution 4.0 International license.

Plasmids p1532-MoMKA1 and p1532-MoMKA1-GFP were constructed with fragment recombination *in vitro*. Fragments were amplified with primer pairs MKA1-1532-F/MKA1-1532-R and MKA1-GFP-F/MKA1-GFP-R and then ligated into vectors pCB1532 and pCB1532-GFP, respectively, using the ClonExpress II reaction system (Vazyme Biotech Co., Ltd., China). The plasmids were introduced into the *ΔMomka1* mutant, and transformants were screened for sulfonylurea resistance (67).

### Y2H assays.

The yeast two-hybrid (Y2H) assays were performed according to the BD Matchmaker library construction & screening kit instructions (Clontech, Palo Alto, CA). The plasmids used in the Y2H assays were constructed using the ClonExpress II reaction system (Vazyme Biotech Co., Ltd., China). The resulting vectors were cotransformed into yeast strain Y2H Gold following the lithium acetate/single-stranded carrier DNA/polyethylene glycol (LiAc/SS-DNA/PEG) transformation protocol. The synthetic defined medium (SD)-Leu-Trp yeast transformants were isolated and assayed for growth on SD-Ade-Leu-Trp-His plates at 30°C for 3 days.

### Immunoblot and co-IP assays.

For immunoblot analysis, total proteins were extracted from vegetative hyphae as previously described (69). Protein samples were fractioned by SDS-PAGE gels and then transferred to a polyvinylidene difluoride (PVDF) membrane and immunoblotted with primary antibody (anti-GFP antibody, Abmart; anti-Flag antibody, Abmart; anti-Pmk1 antibody, Cell Signaling Technology; anti-TpEY antibody, Cell Signaling Technology; anti-Atg13 antibody, Beyotime Biotechnology; and anti-glyceraldehyde-3-phosphate dehydrogenase [GAPDH] antibody, Huabio) at the recommended dilutions. HiSec horseradish peroxidase (HRP)-conjugated goat anti-mouse IgG (H+L) (Vazyme Biotech Co., Ltd., China) and HRP-conjugated goat anti-rabbit IgG (Hangzhou HuaAn Biotechnology Co., Ltd., China) were used as secondary antibodies. The FD FDbio-Femto Rcl kit (Fdbio Science, China) was used to detect the chemiluminescent signals (70).

For immunoprecipitation assays, the *MoMKA1-GFP*, *MST50-3×FLAG*, *MoATG13-3×FLAG*, and *MoATG6-3×FLAG* plasmids were constructed as described above. Total proteins were isolated and incubated with GFP Nanoselector agarose beads (Hangzhou HuaAn Biotechnology, China) or anti‐Flag M2 affinity gel (Sigma) according to the manufacturer’s instructions. Proteins eluted from beads were detected by immunoblot analysis with anti-Flag or anti-GFP antibodies.

### RNA isolation and qRT-PCR analyses.

Total RNA was isolated from mycelium and appressoria using the PureLink RNA minikit (Invitrogen, USA), and then the first strand of cDNA was synthesized with reverse transcription (RT) using HiScript II Q RT SuperMix for qPCR (+gDNA wiper) (Vazyme Biotech Co., Ltd., China). Quantitative RT-PCR (qRT-PCR) was performed with ChamQ SYBR qPCR master mix (Vazyme Biotech Co., Ltd., China) using the Bio-Rad CFX96 real-time system. The primers used to amplify *MoMKA1*, *MoATG13*, and *GAPDH* in qRT-PCR assays are listed in Table S1. The relative quantity of each transcript was calculated by the 2^−ΔΔ^*^CT^* method (71). All qRT-PCRs were conducted in three replicates for each sample, and three biological replicates were maintained.

### Light and fluorescence confocal microscopy.

Light microscopy was used to visualize conidia, appressoria, and penetration of M. oryzae. Fluorescence confocal microscopy was applied to visualize GFP- and CMAC (7-amino-4-chloromethylcoumarin) (Invitrogen, USA)-stained vacuoles. The excitation/emission wavelengths used were 488 nm/507 nm for GFP and 494 nm/518 nm for CMAC.

### Autophagy assay.

To analyze the autophagy process in the Ku80 strain and the *ΔMomka1* mutant, the *GFP-MoATG8* construct was transformed into protoplasts of Ku80 and the *ΔMomka1* mutant. The GFP-MoAtg8-expressing strains were cultured in CM liquid medium at 28°C for 48 h and then transferred to MM-N liquid medium for 6 h and 12 h. The harvested mycelia were subjected to proteolysis assay using anti-GFP antibody. Besides this, hyphae in CM medium were stained with CMAC and visualized by fluorescence confocal microscopy.
