# Supplementary figures and images for: The Paxillin MoPax1 Activates Mitogen-Activated Protein (MAP) Kinase Signaling Pathways and Autophagy through MAP Kinase Activator MoMka1 during Appressorium-Mediated Plant Infection by the Rice Blast Fungus Magnaporthe oryzae
Source: mBio. 2022 Oct 31;13(6):e02218-22. doi: 10.1128/mbio.02218-22 (PMC9765475; doi:10.1128/mbio.02218-22)

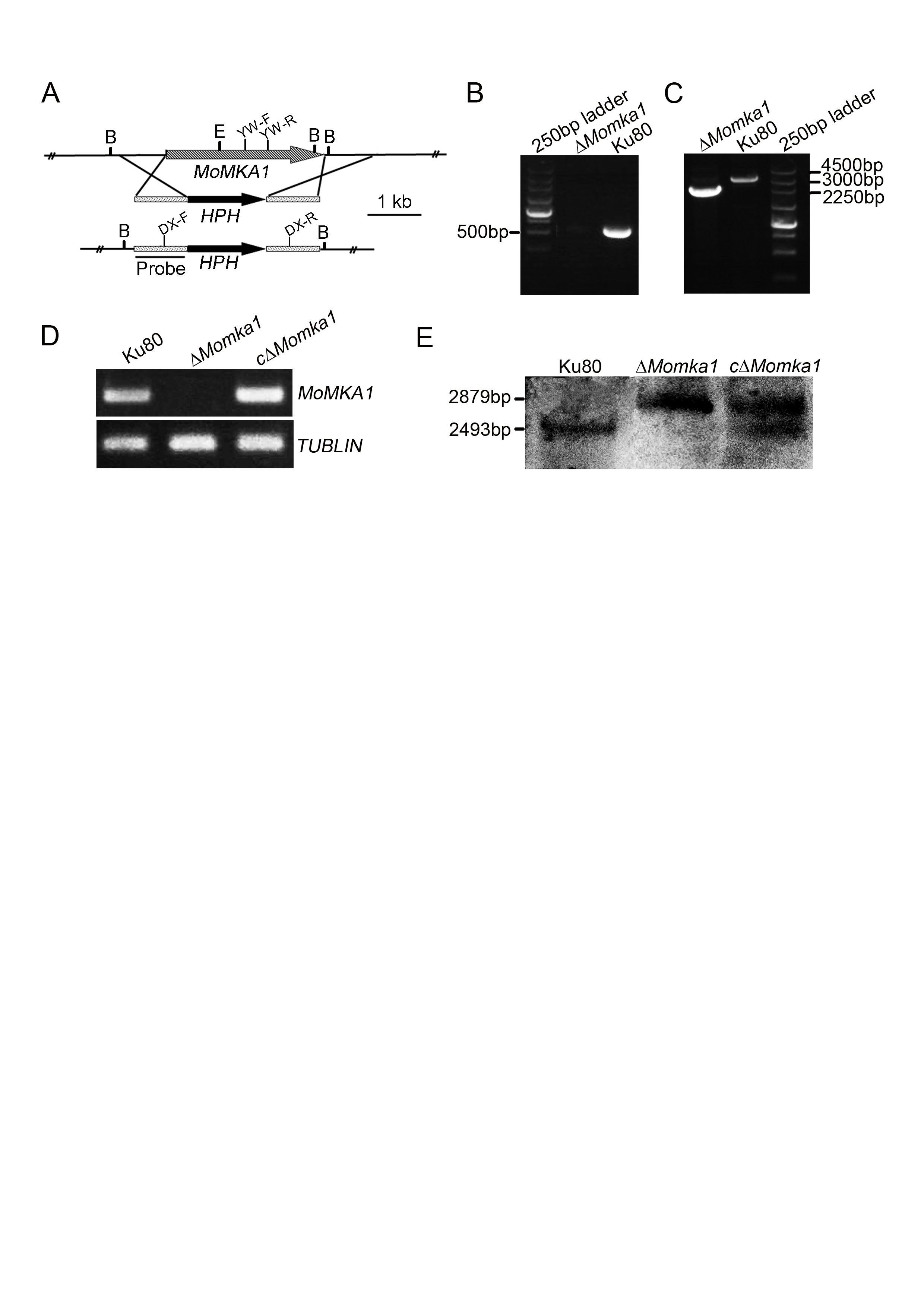

Supplement: FIG S1 [file mbio.02218-22-s0001.tif]

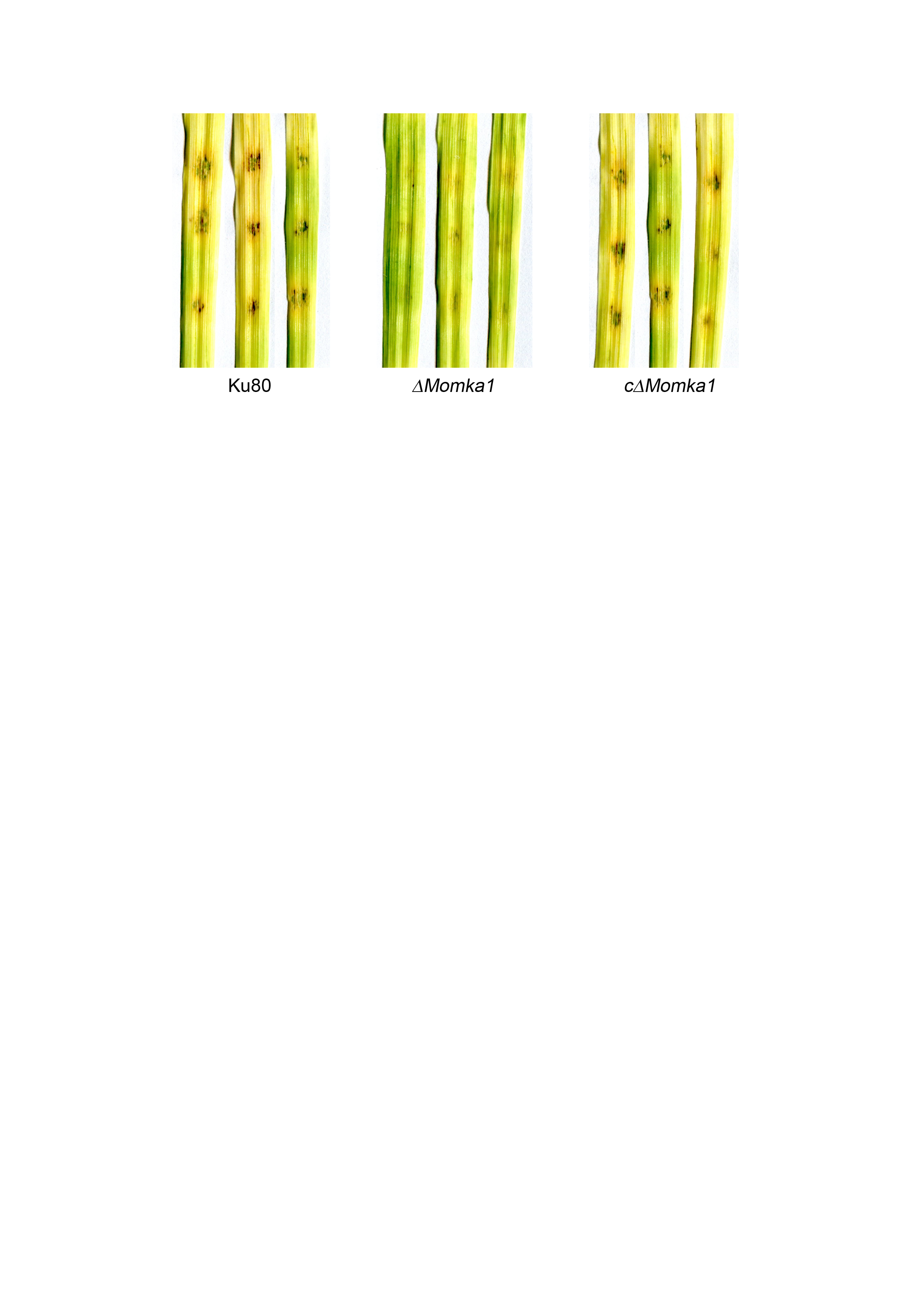

Supplement: FIG S2 [file mbio.02218-22-s0002.tif]

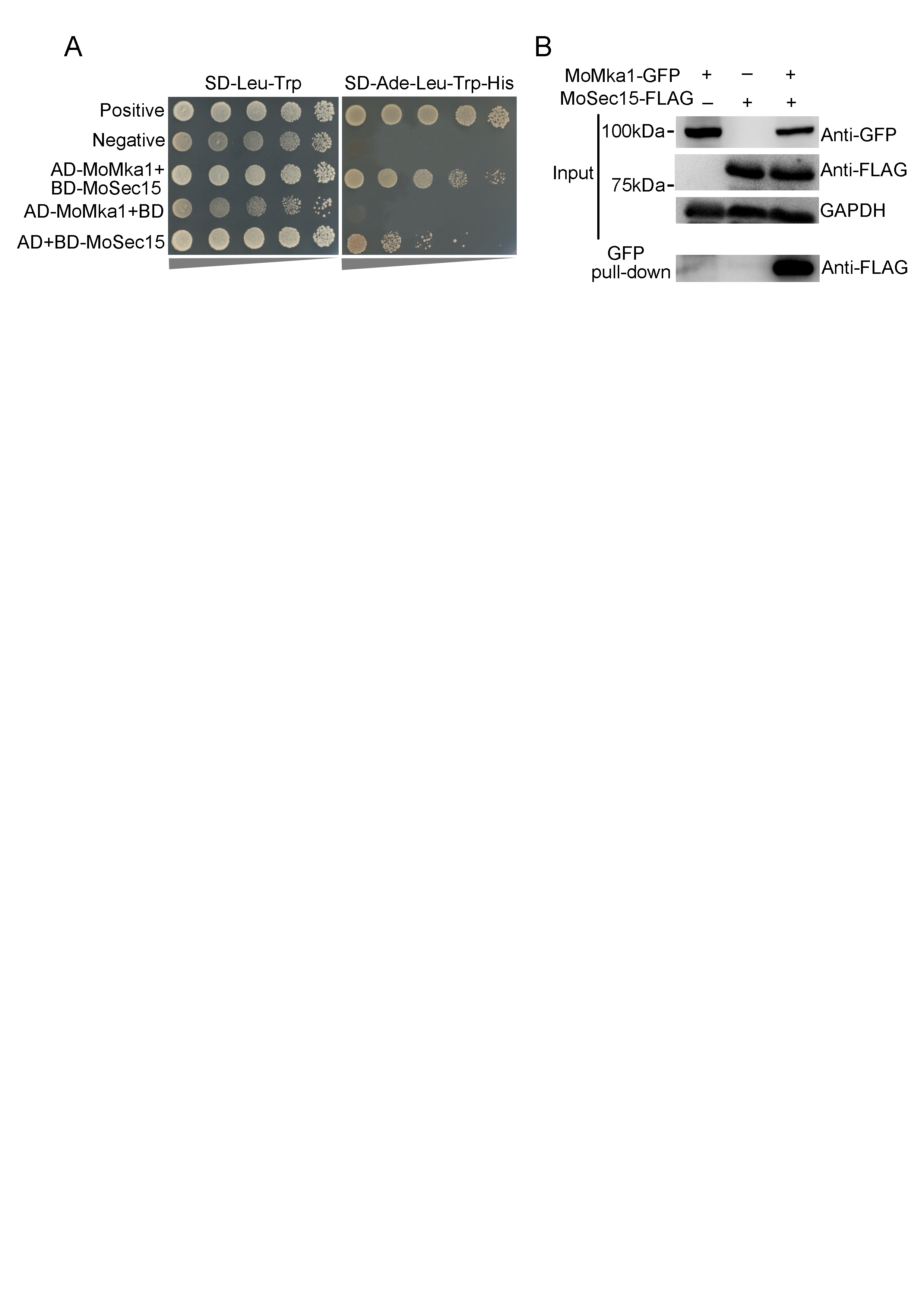

Supplement: FIG S3 [file mbio.02218-22-s0003.tif]

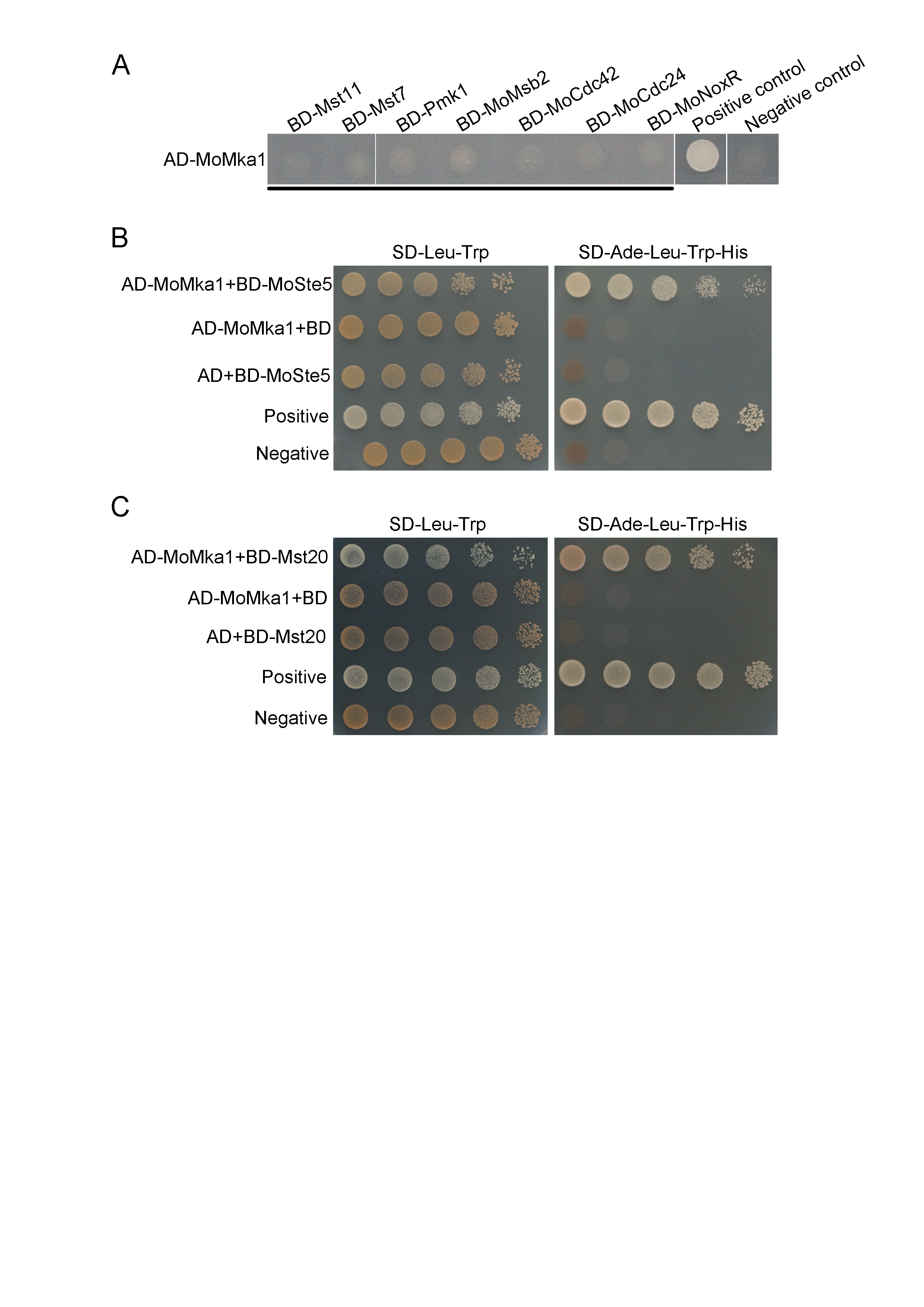

Supplement: FIG S4 [file mbio.02218-22-s0004.tif]
