# Supplementary material for: The Paxillin MoPax1 Activates Mitogen-Activated Protein (MAP) Kinase Signaling Pathways and Autophagy through MAP Kinase Activator MoMka1 during Appressorium-Mediated Plant Infection by the Rice Blast Fungus Magnaporthe oryzae
Source: mBio. 2022 Oct 31;13(6):e02218-22. doi: 10.1128/mbio.02218-22 (PMC9765475; doi:10.1128/mbio.02218-22)
Supplement: TABLE S1 [file mbio.02218-22-s0005.docx]

**Table S1. Primers used in this study.**

| Primer | Sequence (5’-3’) | Relevant characteristics |
| --- | --- | --- |
| UP-F | CCCCCGGGCTGCAGGAATTCACGGTCGGTTGGTAAGGT | Amplification of upstream fragment of *MoMKA1* for the gene deletion |
| UP-R | GCTCCTTCAATATCATCTTCTCTCGTGACGCGAGTTGCAGATT |  |
| Down-F | TAGAGTAGATGCCGACCGAACAAGAGACGCAAATGGAGAAGAA | Amplification of downstream fragment of *MoMKA1* for the gene deletion |
| Down-R | TACCGGGCCCCCCCTCGAGACGCCGATGCCGCCTAAT |  |
| MKA1-1532-F | TCCCCCGGGCTGCAGGAATTCGTAGCAAGGTAGCAGGTT | Amplification of fragment including full length ORF of *MoMKA1* and its native promoter and terminator DNA sequences for the complementation |
| MKA1-1532-R | GATAAGCTTGATATCGAATTCCGTCCCTTGGACACTAAAT |  |
| MKA1-GFP-F | GGATCCCCCGGGCTGCAGGAATTCGTAGCAAGGTAGCAGGTT | Amplification of the native promoter region and full length ORF of *MoMKA1* for fusion with GFP tag |
| MKA1-GFP-R | CAGCTCCTCGCCCTTGCTCACCATTATGTGATCAACGTAGAACA |  |
| AD-F | CATATGGCCATGGAGGCCAGTGAATTCATGAAGGCCTTACGGCGATC | Amplification of the full-length cDNA sequence of *MoMKA1* for fusion with pGADT7 plasmid |
| AD-R | TCGATGCCCACCCGGGTGGAATTCTTATATGTGATCAACGT |  |
| PAX1-BK-F | CATATGGCCATGGAGGCCGAATTCATGTTTGCTCGAGGGAAGTC | Amplification of the full-length cDNA sequence of *MoPAX1* for fusion with pGBKT7 plasmid |
| PAX1-BK-R | TCGACGGATCGCCGGGAATTCTCAGCCCTTCAGCTCCC |  |
| PAX1-Flag-F | TGGGTACTCAAATTGGTTCTCGAGAGTCATTGCGGCCAGAG | Amplification of the native promoter region and full length ORF of *MoPAX1* for fusion with FLAG tag |
| PAX1-Flag-R | CGTCATGGTCTTTGTAGTCGCCCTTCAGCTCCCTCG |  |
| Mst50-BK-F | CATATGGCCATGGAGGCCGAATTCATGAGCTTCAACACGGGGAC | Amplification of the full-length cDNA sequence of *MST50* for fusion with pGBKT7 plasmid |
| Mst50-BK-R | TCGACGGATCGCCGGGAATTCTCATATTATTCCTCCTGGGG |  |
| Mst50-Flag-F | TGGGTACTCAAATTGGTTCTCGAGAACCGCTGTCCGTCCATT | Amplification of the native promoter region and full length ORF of *MST50* for fusion with FLAG tag |
| Mst50-Flag-R | CGTCATGGTCTTTGTAGTCTATTATTCCTCCTGGGG |  |
| ATG6-BK-F | CATATGGCCATGGAGGCCGAATTCATGATGTTTTGCCAAA | Amplification of the full-length cDNA sequence of *MoATG6* for fusion with pGBKT7 plasmid |
| ATG6-BK-R | CGACGGATCGCCGGGAATTCTTAGGTCGAGCTTGAGC |  |
| ATG6-Flag-F | TGGGTACTCAAATTGGTTCTCGAGCAGAACCGAACTCTACAACG | Amplification of the native promoter region and full length ORF of *MoATG6* for fusion with FLAG tag |
| ATG6-Flag-R | CGTCATGGTCTTTGTAGTCGGTCGAGCTTGAGCCCAAAAC |  |
| ATG13-BK-F | CATATGGCCATGGAGGCCGAATTCATGCATCAACAGTCCCGCTA | Amplification of the full-length cDNA sequence of *MoATG13* for fusion with pGBKT7 plasmid |
| ATG13-BK-R | TCGACGGATCGCCGGGAATTCTCACCAGCCTCGCCTTGTG |  |
| ATG13-Flag-F | GGGTACTCAAATTGGTTCTCGAGATCGCCCAAATCACAACA | Amplification of the native promoter region and full length ORF of *MoATG13* for fusion with FLAG tag |
| ATG13-Flag-R | CGTCATGGTCTTTGTAGTCCCAGCCTCGCCTTGTGATTC |  |
| SEC15-BK-F | CATATGGCCATGGAGGCCGAATTCATGCCGCGGAAGCCACAGTC | Amplification of the full-length cDNA sequence of *MoSEC15* for fusion with pGBKT7 plasmid |
| SEC15-BK-R | TCGACGGATCGCCGGGAATTCTCAGCTGAAACCAAAACGAG |  |
| MKA1-RT-F | TCCTGACCGAGTTCCCTG | Amplification of the fragment using for qRT-PCR to detecting the expression level of *MoMKA1* |
| MKA1-RT-R | GCGAAGATTGTTGTGACCC |  |
| P-F | ACGGTCGGTTGGTAAGGT | Amoplification of the fragment using for southern blot to confirm the deletion of *MoMKA1* |
| P-R | CGTATGTTCGGCGTCAG |  |
| ATG13-RT-F | GGAACCCGTGCCTGATTT | Amplification of the fragment using for qRT-PCR to detecting the expression level of *MoATG13* |
| ATG13-RT-R | GCCACTACCGCCATACCTC |  |
